# Mitochondrial Peroxiredoxin III is a Potential Target for Cancer Therapy

**DOI:** 10.3390/ijms12107163

**Published:** 2011-10-21

**Authors:** In-Sung Song, Hyoung-Kyu Kim, Seung-Hun Jeong, Sung-Ryul Lee, Nari Kim, Byoung Doo Rhee, Kyung Soo Ko, Jin Han

**Affiliations:** National Research Laboratory for Mitochondrial Signaling, Department of Physiology, College of Medicine, Cardiovascular and Metabolic Disease Center, Inje University, Busan 614-7-5, Korea; E-Mails: microvirus@hanmail.net (I.-S.S.); estrus74@gmail.com (H.-K.K.); shjeong96@gmail.com(S.-H.J.); lsr1113@hotmail.com(S.-R.L.); narikim43@gmail.com(N.K.); bdrhee@hanmail.net (B.D.R.); kskomd@paik.ac.kr (K.S.K.)

**Keywords:** ROS, mitochondria, peroxiredoxin III, cancer, antioxidant, oxidative stress, therapy

## Abstract

Mitochondria are involved either directly or indirectly in oncogenesis and the alteration of metabolism in cancer cells. Cancer cells contain large numbers of abnormal mitochondria and produce large amounts of reactive oxygen species (ROS). Oxidative stress is caused by an imbalance between the production of ROS and the antioxidant capacity of the cell. Several cancer therapies, such as chemotherapeutic drugs and radiation, disrupt mitochondrial homeostasis and release cytochrome *c*, leading to apoptosome formation, which activates the intrinsic pathway. This is modulated by the extent of mitochondrial oxidative stress. The peroxiredoxin (Prx) system is a cellular defense system against oxidative stress, and mitochondria in cancer cells are known to contain high levels of Prx III. Here, we review accumulating evidence suggesting that mitochondrial oxidative stress is involved in cancer, and discuss the role of the mitochondrial Prx III antioxidant system as a potential target for cancer therapy. We hope that this review will provide the basis for new strategic approaches in the development of effective cancer treatments.

## 1. Mitochondria and Cancer

As the main energy producers in cells, mitochondria subject substrates to oxidative phosphorylation, thereby generating the energy molecule ATP. During this process, mitochondria inevitably generate reactive oxygen species (ROS). ROS are involved in the regulation of many physiological processes, including cell signaling, but are harmful to cells if produced in excessive amounts. Furthermore, mitochondria, which are crucial regulators of the intrinsic pathway of apoptosis, perform vital and lethal functions in physiological and pathological contexts [1,2]. Mitochondria control the activation of apoptotic effector mechanisms by regulating the translocation of pro-apoptotic proteins from the mitochondrial intermembrane space to the cytosol. In addition, they play a major role in multiple forms of non-apoptotic cell death [3]. In this context, mitochondrial abnormalities occur in various diseases, including cardiovascular, neurodegenerative, metabolic diseases, and cancer.

In cancer cells, key mitochondrial regulators of cell death and other processes are often altered [4]. Cancer-cell mitochondria differ structurally and functionally from their normal-cell counterparts [4,5]. Rapidly growing tumors readily become hypoxic due to the inability of the local vasculature to supply an adequate amount of oxygen. Furthermore, mutations in mitochondrial and nuclear DNA that affect components of the mitochondrial respiratory chain result in inefficient ATP production, ROS overproduction, and oxidative damage to mitochondria and macromolecules [5]. Over 70 years ago, Warburg pioneered research into alterations in mitochondrial respiration in the context of cancer and proposed a mechanism to explain how they evolve during the carcinogenic process [6]. This process differs from that in normal cells, which utilize oxidative phosphorylation primarily for growth and survival. Although the observation of high rates of aerobic glycolysis in tumor cells has been corroborated, the role of mitochondria in tumor cells has been contentious [7]. The major role of aerobic glycolysis in cancer cells is likely to be the generation of glycolytic intermediates for the pentose phosphate pathway in nucleotide and phospholipid synthesis, while glycolytic ATP generation is likely to be important for survival under hypoxic conditions [8]. The glutamine-fueled TCA cycle generates ATP, ROS, nicotinamide adenine dinucleotide phosphate (NADPH), amino acids, and lipids. The synthesis of ATP requires large amounts of oxygen, which routinely leads to the generation of ROS such as hydrogen peroxide, the superoxide anion, and organic peroxide [9]. These ROS can cause cellular damage if they are not detoxified by antioxidant systems. Increased mitochondrial ROS generation and the disturbance of peroxiredoxin (Prx) production in cancer cells may lead to oxidative stress and the induction of apoptosis. The Prx system is a cellular defense system against oxidative stress. Mitochondria in cancer cells are known to contain high levels of Prx III and Prx V [10–14]. However, Prx V founds in various compartments in the cell, including mitochondria, peroxisome and nucleus [15–17]. Moreover, mitochondria are a major site of hydrogen peroxide generation in cells [18]. Prx III prefers to scavenge hydrogen peroxide, which will be the target for up to 90% of H_2_O_2_. In contrast, Prx V behaves more effectively as a scavenger of peroxynitrite [19–22]. Here, we discuss the role of the mitochondrial Prx III antioxidant system being exclusively present in mitochondria as a potential target for cancer therapy, and examine the effects of antioxidant proteins on ROS in mitochondria. We hope that this review article will advance our understanding of mitochondrial biology in cancer, and provide a basis for designing new strategies to achieve effective cancer treatment.

## 2. Mitochondrion-Targeting Cancer Therapy

**Figure 1:**
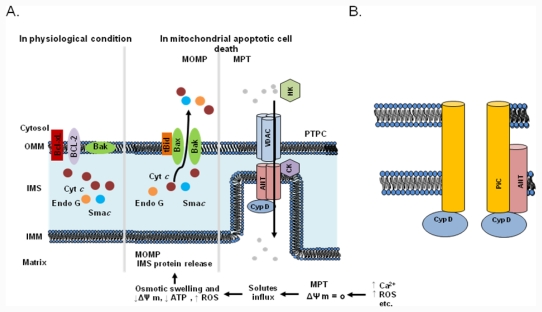
Molecular mechanisms of the mitochondrial permeability transition (MPT) and mitochondrial apoptotic cell death. (a) Mitochondrial outer membrane permeabilization (MOMP) leads to apoptogenic protein release. Bax or Bak forms a pore in the OM after activation by a BH3-only protein such as Bid (after the truncation of Bid by caspase-8). The opening of the PT pore allows an influx of water and ions into the matrix, causing matrix swelling. This leads to rupture of the OM and the release of intermembrane space (IMS) proteins. The permeability transition pore complex (PTPC) is a highly dynamic supramolecular entity that can comprise a voltage-dependent anion channel (VDAC), adenine nucleotide translocase (ANT), and cyclophilin D (CypD). Other proteins, including the peripheral benzodiazepine receptor (PBR), hexokinase (HK), and creatine kinase (CK), may also be associated with the PTPC. It is not clear whether the PTPC has a role under physiological conditions. Mitochondria exhibit a high mitochondrial transmembrane potential, which is generated by the respiratory chain and exploited for ATP generation. It has been proposed that under these conditions the PTPC exists in a low-conductance state, thereby contributing to the exchange of small metabolites between the cytosol and the mitochondrial matrix, a process that is predominantly mediated by mitochondrial solute carriers. However, under pathological conditions characterized by a high Ca^2+^ concentration, increased oxidative stress, low levels of ATP, and mitochondrial depolarization, the complex forms an open pore between the inner and outer membranes, allowing the free diffusion of solutes across the membranes. The opening of the PTPC results in mitochondrial swelling, mitochondrial Ca^2+^ efflux, and the release of apoptogenic proteins such as cytochrome *c* and Sma*c* from the IMS. (b) Alternative models proposed in light of recent findings in gene-targeted mice. A VDAC is no longer part of the model and it appears that an OM component may not be necessary for this process. ANT now appears to be more of a regulatory protein, and only CypD remains as an established component. In contrast, the mitochondrial phosphate carrier (PiC) has been added to the model as a candidate component of the pore-forming unit of the MPT pore.

Mitochondria are known to play a key role in apoptosis and to trigger cell death via several mechanisms, including the disruption of electron transport and energy metabolism, the release or activation of proteins that mediate apoptosis, and the alteration of the cellular redox potential [23–25]. Apoptotic cell death is characterized by a host of morphological and biochemical features, including mitochondrial outer membrane permeabilization (MOMP) and the release of pro-apoptotic proteins [26]. In response to pro-apoptotic stimuli, including ROS and Ca^2+^ overload, the permeability transition pore complex (PTPC) assumes a high-conductance state that deregulates the entry of small solutes into the mitochondrial matrix along their electrochemical gradients[1]. This mitochondrial permeability transition (MPT) results in immediate dissipation of the mitochondrial membrane potential and osmotic swelling of the mitochondrial matrix. As the surface area of the inner membrane considerably exceeds that of the outer membrane, the MPT eventually leads to MOMP (Figure 1). The MPT can be triggered by agents that increase cytosolic Ca^2+^ concentrations or stimulate ROS generation. The mitochondrial pore, a putative multimeric complex situated at mitochondrial contact sites, mediates the MPT. Based on biochemical evidence, the standard model for the PT pore consists of a voltage-dependent anion channel (VDAC) in the outer membrane, adenine nucleotide translocase (ANT), and cyclophilin D (CypD) in the matrix (Figure1A; [27,28]. As shown in Figure 1A, the VDAC has always been considered a key component of the PTPC. However, considerable recent evidence suggests that the conclusions of standard model studies were incorrect (Figure 1B). Closure of the VDAC has been shown to increase the influx of Ca^2+^ into mitochondria [29], which has the net effect of inducing, rather than inhibiting, the MPT. Moreover, recent genetic studies have confirmed a regulatory role for CypD in the MPT [30,31]. Mice lacking ANT or the VDAC still exhibit a classical MPT response that is inhibited by cyclosporine A [32,33]. Thus, current genetic strategies indicate that only CypD functions as a necessary effector of the MPT, and suggest that alternative proteins and/or mechanisms must play roles in mitochondrial-dependent cell mortality via the PT pore. Because two proposed models for the PT pore have not yet been fully elucidated, the PT pore is not sufficiently well characterized to be a target for anticancer drugs. Although the exact molecular identity of the effectors of the MPT is under debate, it is agreed to be a crucial step in cell death.

Because most cancer cells have increased resistance to the activation of MOMP and escape apoptosis as a result of various modifications in apoptosis regulators, including Bcl-2 family members, p53, and caspases [34], various mitochondrion-targeted cancer treatment strategies have been developed in the last decade [35,36]. These strategies focused mainly on the development of compounds that regulate mitochondrial Bcl-2 family proteins, modulate MOMP and hyperpolarized mitochondria inner membrane potential sensing, or target high levels of ROS and overexpressed receptors in cancer cells [35]. An excellent previous review by Fulda *et al.* summarized examples of mitochondrion-targeted compounds (Table 1); [36]. Numerous molecules that are currently in use or being tested in clinical trials act on mitochondria [37]. Clinically approved anticancer drugs such as etoposide [38], paclitaxel [39], and vinorelbine [40], as well as an increasing number of experimental anticancer drugs, including ceramide [41], MKT077 [42], and CD437 [43], have been found to act directly on mitochondria to trigger apoptosis. Several classes of compounds with distinct mechanisms of action can stimulate the MPT and mitochondrial apoptosis in cancer cells, pointing to some functional redundancy and suggesting the likely existence of alternative biochemical cascades leading to mitochondrial membrane permeabilization. Thus, the selective targeting of cancer cells using mitochondrial-targeted agents is likely to attract great interest. A better understanding of the key pathophysiological differences between mitochondria in cancer cells and their counterparts in non-cancerous cells will undoubtedly be instrumental in increasing the level of selectivity of mitochondrion-targeted anticancer agents. Nevertheless, a limited number of studies have evaluated agents targeting the mitochondrial ROS regulatory system.

## 3. Regulation of the Mitochondrial Antioxidant System

**Figure 2:**
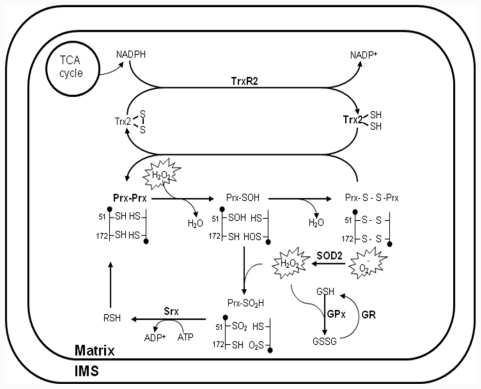
Antioxidant system for H_2_O_2_ removal in mitochondria. Reactive oxygen species (ROS), in the form of O_2_^−^ and H_2_O_2_, have multiple intra- and extramitochondrial sources. O_2_^−^ is converted to H_2_O_2_ through the action of superoxide dismutase (SOD) 2 and /or spontaneous dismutation. H_2_O_2_ can diffuse into the mitochondrial matrix, where it is removed via three systems/mechanisms: 1) peroxiredoxin (Prx) III coupled to thioredoxin (Trx) 2 and Trx reductase (TrxR) 2; 2) glutathione peroxidase (GPx) coupled to glutathione (GSH) and GSH reductase (GR); and 3) non-enzymatic scavenging by redox compounds. The peroxidatic cysteine Cys-SH is selectively oxidized by H_2_O_2_ to Cys-SOH, which then reacts with the resolving cysteine Cys-SH of the other subunit in the homodimer to form an intermolecular disulfide bond. Subsequently, the disulfide bond is specifically reduced by Trx2, which in turn receives reducing equivalents from nicotinamide adenine dinucleotide phosphate (NADPH) via TrxR2. The Cys-SOH generated is oxidized to Cys-SO_2_H, leading to peroxidase inactivation. Reactivation of the enzyme is achieved by reduction of the Cys-SO_2_H moiety in a reaction that requires ATP hydrolysis and is catalyzed by sulfiredoxin (Srx), with reducing equivalents provided by physiological thiols (RSH) such as GSH and Trx. The respiration substrates malate/glutamate and succinate provide energy in the form of reducing covalents (NADPH), which are maintained by ΔΨm-dependent transhydrogenase and tricarboxylic acid (TCA) cycle enzymes. NADPH is utilized by the reductases in the peroxidase system (TrxR and GR) to reduce disulfide bonds formed in proteins during the detoxification of H_2_O_2_.

Since the discovery that electron leakage and incomplete reduction of oxygen occur in the respiration chain [44], mitochondria have been considered a major contributor to cellular oxidative damage due to their generation of ROS. Moreover, mitochondria possess a multilevel network of enzymatic and non-enzymatic antioxidant systems for the detoxification of H_2_O_2_ (Figure 2). The biological significance of mitochondrial ROS has been highlighted by the targeted deletion or overexpression of antioxidant proteins. For example, superoxide dismutase (SOD) 2, thioredoxin (Trx) 2, Prx III and Prx V have been reported to constitute a novel antioxidant defense system that detoxifies ROS generated in mitochondria [45,46]. Prx3-knockout (KO) mice were showed aberrant regulation of oxidative stress. Proteomic analysis and gene expression analysis in adipocytes from Prx3-KO mice also showed defect in mitochondria biogenesis along with enzymes involved in glucose/lipid metabolism and oxidative phosphorylation [47]. Trx2-KO mice have an embryonic lethal phenotype [48]. SOD2-KO mice typically die within 3 weeks of birth as a result of severe neurodegeneration and mitochondrial oxidative damage [49,50]. Prx V was associated with the mitochondrial pathway of apoptosis and calcium loading capacity of mitochondria, as well as changes in mitochondrial morphology [14]. The homozygous glutathione peroxidase (GPx)1-KO mice appeared healthy and manifested no increased sensitivity to hyperoxia or increased levels of protein carbonyl groups or lipid peroxides [51]. However, a protective role for GPx1 became apparent, when the GPx1 KO and control mice were subjected to extreme oxidative stress such as that associated with ischemia-reperfusion injury or treatment with paraquat or a bolus of H_2_O_2_ [52,53]. In mammalian cells, GPx1 is the major isoform and is expressed in all tissues; it is localized predominantly in the cytosol, but a small proportion (10%) of GPx1 molecules is also present in the mitochondrial matrix [51,54,55]. Thereby, it remains unclear whether the effect of GPx1-KO under these conditions was attributable to the absence of the enzyme form the cytosol or from mitochondria, or from both. To date, the multiplelevel network of antioxidant system in mitochondria has been extensively discussed in a number of recent publications. Based on these studies, mitochondrial-targeted agents emerge as a means to selectively target tumors. Here, we provide a comprehensive compendium on the mitochondrial-targeted compounds for the treatment of human cancer.

Multiple compounds act on components of the antioxidant system to induce ROS generation and apoptosis. Reportedly, the increase in intrinsic ΔΨm correlates with increased malignancy (apoptosis resistance and tumor progression) [56], suggesting that cytotoxic agents that permeabilize the mitochondrial membrane, such as compounds that induce the overproduction of ROS, are effective anticancer drugs in cancer cells. The inhibition of antioxidant systems is an alternative way to induce ROS accumulation. Compounds that inhibit antioxidant systems include the SOD inhibitors 2-methoxyestradiol, choline tetrathiomolybdate (ATN-224), and mangafodipir; buthionine sulfoximine, imexon, and phenylethyl isothiocyanate (PEITC), which cause glutathione (GSH) inhibition or depletion; and menadione, motexafin gadolinium, β-lapachone, elesclomol (STA-4783), arsenic trioxide, parthenolide, dimethylamino-parthenolide (DMAPT), and bistetrahydrofuranic acetogenins, which induce ROS production (Table 2).

2-methoxyoestradiol inhibits angiogenesis by reducing endothelial cell proliferation and inducing endothelial cell apoptosis, and selectively kills human leukemia cells by inhibiting SOD, thereby causing superoxide accumulation [57]. Several Phase I/II trials in patients with solid malignancies or multiple myeloma have demonstrated that 2-methoxyoestradiol is well tolerated and causes disease stabilization [58–60]. Similar effects are produced by the intracellular copper-chelating agent ATN-224 [61]. ATN-224 is an orally bioavailable, second-generation tetrathiomolybdate analog with potential antiangiogenic and antineoplastic activities. Mangafodipir is a SOD mimic with catalase and GSH reductase activities. Consisting of manganese ions chelated to fodipir (dipyridoxyl diphosphate; DPDP), it scavenges oxygen free radicals such as the superoxide anion, hydrogen peroxide, and the hydroxyl radical, potentially preventing oxygen free radical damage to macromolecules such as DNA and minimizing oxygen free radical–related chemotoxicity in normal tissues. In cancer cells, it has been shown to increase H_2_O_2_ levels and to potentiate the antitumor activity of paclitaxel in a mouse xenotransplant colon cancer model [62]. Moreover, it is being tested in a Phase II trial in patients with colon cancer.

Buthionine sulfoximine irreversibly inhibits γ-glutamylcysteine synthetase. It increases ROS levels by inhibiting the synthesis of reduced GSH [63]. Imexon depletes the GSH pool due to its thiol-binding activity [64]. Buthionine sulfoximine and the alkylating agent melphalan are being evaluated in Phase II clinical trials in patients with melanoma or relapsed/refractory ovarian cancer. PEITC, which is thiol modifier, preferentially causes ROS overproduction, mitochondrial oxidative damage, MOMP, and apoptosis in cancer cells, presumably due to their increased ROS levels [65,66]. The compound is known effects on the selenoprotein thioredoxin reductase, glutathione reductase and intracellular GSH levels. Moreover, Prx III is early oxidized after exposure of this compound [67].

Menadione binds to and inhibits the activity of the PTPs that dephosphorylate and inactivate epidermal growth factor receptor (EGFR) and erythroblastic leukemia viral oncogene homolog 2 (ErbB2) in human keratinocytes. Local reversal of EGFR and ErbB2 inhibition associated with the systemic administration of EGFR inhibitors may help alleviate EGFR inhibitor–mediated skin toxicity. Menadione undergoes futile redox cycles in the respiratory chain. Thiol cross-linking agents, such as diamide, bismaleimido-hexane, and dithiodipyridine, cause ANT thiol oxidation and can bypass B-cell lymphoma 2 (BCL-2)–mediated cytoprotection [78,79]. β-lapachone is bioactivated by NAD(P)H:quinone oxidoreductase-1 (NQO1), causing futile oxidoreduction that generates high levels of superoxide, and is currently under clinical investigation, as a monotherapy or in combination with gemcitabine, in patients with pancreatic and head-and-neck cancer. STA-4783 induces oxidative stress, increasing levels of ROS such as hydrogen peroxide in both cancer cells and normal cells. Because tumor cells have elevated levels of ROS compared with normal cells, the increase in oxidative stress beyond baseline levels elevates ROS levels beyond sustainable levels, exhausting tumor cell antioxidant capacity. This may result in the activation of the mitochondrial apoptosis pathway [72]. Arsenic trioxide is a small-molecule arsenic compound with antineoplastic activity. Although the mechanism of action of arsenic trioxide is not completely understood, it causes damage to or degradation of the promyelocytic leukemia protein/retinoic acid receptor-α (PML/RARα) fusion protein; induces apoptosis in acute promyelocytic leukemia cells and many other tumor cell types; promotes cell differentiation and suppresses cell proliferation in many different tumor cell types; and is pro-angiogenic. Parthenolide is a sesquiterpene lactone that can cause allergic reactions. It has anti-inflammatory, antimicrobial, and anticancer properties, activates the tumor suppressor p53, and inhibits nuclear factor-kappa B (NF-κB) and the signal transducer and activator of transcription 3 (STAT-3; [80]. It also induces intracellular oxidative stress, which is manifested by increased ROS levels and activation of c-Jun N-terminal kinase (JNK). The water-soluble parthenolide analog DMAPT, which swiftly kills leukemic stem cells from both myeloid and lymphoid leukemias, is also highly cytotoxic to bulk leukemic cell populations. Molecular studies have found that the key activities of DMAPT include the induction of oxidative stress responses, the inhibition of NF-κB, and the activation of p53 [75]. Natural bistetrahydrofuranic acetogenins show growth inhibitory activity against human breast, lung, liver, and colon cell lines [77]. Recently, structure–activity relationship (SAR) analysis has led to the synthesis of promising new derivatives with improved antitumor properties. However, trials of numerous ROS-regulating compounds, including menadione and STA-4783, have been discontinued due to safety concerns.

## 4. Peroxiredoxin III: A Potential Mitochondrial Target for Cancer Therapy

Peroxiredoxins are a family of enzymes that catalyze the reduction of hydrogen peroxide and hydroperoxides to water and alcohol, respectively [81,82]. The six isoforms of mammalian Prx (I–VI) are classified into three subfamilies (2-Cys, atypical 2-Cys, and 1-Cys) based on the number and position of the cysteine (Cys) residues that participate in catalysis. Also, the Prxs can be categorized by their subcellular localization; Prx I, II and VI found in the cytoplasm, Prx IV in the endoplasmic reticulum, Prx III in the mitochondria, and Prx V found in various compartments in the cell, including peroxisomes and mitochondria. Prx I–IV (2-Cys Prx subfamily) have two conserved Cys residues. In the catalytic cycle of the 2-Cys Prxs, the conserved N-terminal Cys sulfhydryl (Cys-SH) is first oxidized by peroxides to Cys sulfenic acid (Cys-SOH), which then reacts with the conserved COOH-terminal Cys-SH of the other subunit in the homodimer to form a disulfide bond. The Prx V is an atypical 2-Cys Prx that becomes oxidized at the peroxidatic cysteine (Cys48) to a sulfenic acid, which condenses with a resolving cysteine (Cys152) within the same polypeptide to form an intramolecular disulfide linkage [16]. In contrast, Prx VI has only one Cys residue is involved in the peroxidase activity Prx VI (1-Cys), and unlike the other members, does not use thioredoxin as a reductant. The N-terminal Cys-SH of Prx VI is readily oxidized, but the resulting Cys-SOH does not form a disulfide because of the unavailability of another Cys-SH nearby [83,84]. As the physiological reductant, Prx VI utilizes GSH via the formation of disulfide with GSH mediated by πGST.

The hyper-proliferative property of cancer cells is known to be associated with increased production of intracellular ROS [85]. Moreover, many reports have claimed an association between alterations in the protein level of Prx isoforms. Such Prxs serve divergent functions, such as protecting cells against oxidative stress, regulating cell signaling associated with H_2_O_2_, and influencing cell differentiation and proliferation, immune responses, and apoptosis [82,86,87] Recent studies reported elevated expression of Prx I in several human cancers, including non-small cell lung cancer (NSCLC) [88,89], oral cancer [90], breast cancer [11], and liver cancer [91]. Prx II levels are increased in breast, mesothelioma, and head-and-neck cancers [10,92]. While increased Prx II expression rendered leukemia and stomach cancer cells resistant to various chemotherapeutic agents [93,94], downregulation of Prx II sensitized head-and-neck cancer cells to radiation and gastric carcinoma to cisplatin [95,96]. Moreover, downregulation of Prx II enhances apoptotic cell death induced by tumor necrosis factor (TNF)-α and TNF-related apoptosis-inducing ligand (TRAIL). Importantly, cytosolic Prx II regulates caspase-8 activation, but exerts no influence on sustained JNK activation [97]. Downregulation of Prx I was shown to sensitize lung cancer cells to radiation and reduce metastasis [98,99], and to increase the sensitivity of prostate cells to androgen ablation treatment [100]. Prx IV is decreased in stomach cancers [101]; may play an important role in protecting cells from ionizing radiation-induced apoptosis in head-and-neck squamous cell carcinoma [102]; in lung cancer cells, Prx IV interacts with surfiredoxin and the interaction axis leads to acceleration of tumor growth and metastasis formation *in vivo* [103]. Prx V represented antioxidant functions in the lung cartilage, and brain [104–106]. Overexpression of Prx V was reported to protect Chinese hamster ovary cells from oxidative stress; suppressed p53-dependent apoptosis [107]; promoted differerentiation, and reduced apoptosis in the mice muscle cells [108] and human tendon cells [109]. However, it still remains unknown whether the function of this protein is restricted to its antioxidant activity, and position of major compartments to protect cells from cell death. Prx VI is decreased in a mouse that is susceptible to experimental atherosclerosis [110] and is elevated in the spinal cord of mice expressing mutant superoxide dismutase1 [111]; in brains of patients with parkinsonian dementia [112], sporadic Creutzfeldt-Jacob disease [113], and Pick disease [114]; in the healing edge of skin wounds [115]; and in experimental cellular premature senescence [116]. Especially, it is elevated in lungs with malignant mesothelioma [10] or high grade squamous cell carcinoma [117].

Like cytosolic Prx I and Prx II, mitochondrial Prx III is overexpressed in hepatocellular carcinoma [12] and breast cancer [11]. The overexpression of Prx III can protect cells against oxidative injury [13,118], whereas the deletion of Prx III in HeLa cells can increase intracellular levels of H_2_O_2_ and sensitize cells to the induction of apoptosis by staurosporine and TNF-α [119]. Furthermore, the abundance of Prx III was found to be reduced in the brains of patients with Alzheimer’s disease and Down syndrome, possibly rendering the neuronal cells of these patients more vulnerable to cell death [120].

The role of Prx III in the scavenging of mitochondrial H_2_O_2_ has recently been emphasized. Originally cloned from murine erythroleukemia cells, Prx III has been identified as a gene induced by oncogenic c-Myc [121]. Its specific localization to mitochondria [122,123] suggests that Prx III, together with its mitochondrion-specific electron suppliers Trx2 and Trx reductase (TrxR) 2 [124,125], might provide a primary line of defense against H_2_O_2_ produced by the mitochondrial respiratory chain [126,127], as SOD2 does against the superoxide radical. In the presence of excess H_2_O_2_, Prx III is highly sensitive to oxidative inaction. Hyperoxidation of Prx III has been observed in cultured cells following prolonged exposure to high levels of H_2_O_2_ or drugs that generate H_2_O_2_ [128–130]. Moreover, hyperoxidized Prx III is reduced more slowly that hyperoxidized Prx I and II in the cytoplasm [129] and the slow reduction will enable the hyperoxidized form of Prx III to accumulate under certain conditions. Therefore, hyperoxidized Prx III formation by H_2_O_2_ leads to an increase in mitochondrial H_2_O_2_ and that this may influence the progression of apoptosis.

In addition, sulfiredoxin (Srx) plays a crucial role by reducing hyperoxidized Prx III via translocation into mitochondria. Noh *et al*. reported that the overexpression of mitochondrion-targeted Srx efficiently promotes the restoration of Prx III and results in cellular resistance to apoptosis, with enhanced elimination of mitochondrial H_2_O_2_ and decreased rates of ΔΨm collapse [131]. Thus, a Trx-related antioxidant system composed of Trx2, TrxR2, and Prx III has been closely associated with the regulation of apoptosis and the redox control of MPT pores for the release of cytochrome *c* [79,94,132]. However, rare attempts to characterize Prx III and its electron suppliers have produced intriguing results that demonstrate the removal of exogenous ROS by actively respiring mitochondria.

## 5. Outlook and Future Perspectives

Most of the currently used cytotoxic anticancer therapeutics have no clear-cut cell specificity, yet tend to kill tumor cells more efficiently than normal cells. With rare exceptions, single drugs at clinically tolerable doses have not been able to cure cancer. Prolonged drug exposure may result in cumulative toxicity. The clinical efficacy of chemotherapy must be enhanced, its attendant toxicity reduced, and resistance overcome. To overcome multidrug resistance in cancer cells, recent chemotherapeutics could be used in combination with other molecules. In the 1960s and early 1970s, drug combination regimens were developed based on the known biochemical actions of available anticancer drugs, rather than on their clinical efficacy. However, such regimens were largely ineffective [133,134]. The era of effective combination chemotherapy began when a number of active drugs of different classes became available for use in combination to treat acute leukemia and lymphomas. After this initial success with hematologic malignancies, combination chemotherapy was applied to the treatment of most solid tumors.

Structural and functional mitochondrial alterations associated with malignant transformation seem to be phenomena common to many types of cancer. Most classical anticancer agents engage signaling pathways that lie upstream of mitochondria and converge on mitochondria due to their role as integrators of pro-death and pro-survival signals. MOMP occurs as a consequence of upstream signaling events that are frequently deregulated in human cancers and that become resistant to a number of conventional therapeutic strategies targeting upstream MOMP regulators. Anticancer drugs that target mitochondria have the potential to bypass the resistance mechanisms that evolved in response to treatment with conventional chemotherapeutics. The combined use of mitochondriontargeted agents with conventional chemotherapeutics and other chemotherapeutic drugs, such as ROS scavenger inhibitors or ROS inducers, may be necessary to achieve maximum efficacy. The pharmacological depletion of ROS scavengers in cancer cells markedly reduces their clonogenicity and results in radiosensitization. As mentioned above, recent studies have shown that the overexpression of Prx III and its electron donors can protect cells, whereas their depletion induced cell death in cancer cells. Therefore, drugs targeting Prx III and the mitochondrion-specific electron suppliers Trx2, TrxR2, and Srx may potentially be administered in combination with various chemotherapeutic agents, including cisplatin, paclitaxel, and etoposide. However, caution must be exercised to prevent a potential increase in toxic side effects. A comprehensive understanding of mitochondrial biology in cancer cells and the interaction between cellular metabolism and drug action is essential in the development of mitochondrion-targeted agents for cancer treatment.

## Figures and Tables

**Table 1:** Examples of mitochondrion-targeted compounds.

Class	Compound	Action(s)/targets
Modulators of the BCL-2 protein family	A-385358	BCL-X_L_
	ABT-263, ABT-737	BCL-2, BCL-X_L_, BCL-W
	AT-101	BCL-2, BCL-X_L_, BCL-W, MCL1
	GX15-070 (Obatoclax)	BCL-2, BCL-X_L_, BCL-W, MCL1
	HA14-1	BCL-2
Metabolic inhibitors	3-bromopyruvate	HK2–VDAC interaction
	Dichloroacetate	PDK inhibition
	HK2 peptide	HK2–VDAC interaction
	LDH-A shRNA	LDH-A
	Methyl jasmonate	HK2–VDAC interaction
	SB-204990	ATP citrate lyase
	Orlistat	Fatty acid synthase
	Soraphen A	Acetyl-CoA carboxylase inhibition
	2-deoxy-*D*-glucose	HK2
VDAC- and/or ANT-targeting agents	Clodronate	ANT inhibition
	GSAO	ANT cross linker
	Lonidamine	ANT ligand
	PK11195	PBR ligand
	Arsenic trioxide	ANT ligand, ROS production
Retinoids	All-*trans*-retinoic acid	ANT ligand
	CD437	Permeability transition pore complex
	ST1926	Perturbation of Ca^2+^ homeostasis
HSP90 inhibitors	Gamitrinibs	Mitochondrial HSP90 ATPase inhibition
	PU24FCI, PU-H58, PU-H71	HSP90 inhibition
	Shepherdin	Inhibition of the HSP90–survivin interaction
Natural compounds and derivatives	α-tocopheryl succinate	Ubiquinone-binding sites in respiratory complex II
	Betulinic acid	Permeability transition pore complex
	Resveratrol	F_1_-ATPase

ANT, adenine nucleotide translocase; BCL-2, B-cell lymphoma protein 2; BCL-W, also known as BCL2-like protein 2 (BCL2L2); BCL-X_L_, also known as BCL2-like protein 1 (BCL2L1); CD437, 6-[3-(1-adamantyl)-4-hydroxyphenyl]-2- naphthalene carboxylic acid; HA14-1, 2-amino-6-bromo-4-(1-cyano-2-ethoxy-2-oxoethyl)-4*H*-chromene-3-carboxylate; GPx, glutathione peroxidase; GSH, reduced glutathione; HK, hexokinase; HSP90, heat shock protein, 90 kDa; LDH-A, lactate dehydrogenase A; MCL1, myeloid cell leukemia sequence 1; PBR, peripheral benzodiazepine receptor; PDK, pyruvate dehydrogenase kinase; PU24FCl, 8-(2-chloro-3,4,5-trimethoxybenzyl)-2-fluoro-9-(pent-4-ynyl)-9*H*-purin-6- amine; PU-H58 (8-(6-bromobenzo[d][1,3]dioxol-5-ylthio)-9-(pent-4-ynyl)-9*H*-purin-6-amine; PU-H71, 8-(6- iodobenzo[d][1,3]dioxol-5-ylthio)-9-(3-(isopropyl amino)propyl)-9*H*-purin-6-amine; ROS, reactive oxygen species; shRNA, short hairpin RNA; SOD, superoxide dismutase; ST1926, (*E*)-3-(4′-hydroxy-3′-adamantylbiphenyl-4-yl)acrylic acid; VDAC, voltage-dependent anion channel. Adapted from [36] with permission.

**Table 2:** Development and clinical status of anti-cancer drugs targeting the mitochondrial oxidative system.

Target	Compound	Action(s)/target(s)	Development status (ClinicalTrials.gov)	Ref.
SOD	2-methoxyestradiol	SOD inhibition	Completed: Phase I in solid tumors	[68,69]
	ATN-224	SOD inhibition	Closed: Phase II in combination with temozolomide in advanced melanoma Closed: Phase II in prostate cancer	[61]
	Mangafodipir	SOD mimic	Active: Phase II in patients who have moderate oxaliplatin neuropathy Completed: Phase II in colon cancer	[62]
GPx	Buthionine sulfoximine (BSO)	GSH synthesis inhibition	Active: Phase I in resistant or recurrent neuroblastoma Completed: Phase II in combination with melphalan in metastatic melanoma and relapsed or refractory ovarian cancer	[63]
	Imexon (Amplimexon)	GSH depletion	Active: Phase II in follicular and aggressive lymphomas Completed: Phase II in multiple myeloma and in combination with gemcitabine in pancreatic cancer Closed : Phase I/II in combination with dacarbazine in stage III and stage IV metastatic melanoma	[64]
	PEITC	GSH depletion, GPx inhibition	Active: Phase II in preventing lung cancer in smokers Phase I in lymphoproliferative disorders Completed: Phase I in preventing lung cancer in smokers	[65]
ROS over-production	Menadione	ROS production	Closed: Phase I in patients treated with EGFR inhibitors	[34]
	Motexafin gadolinium	ROS production	Not yet open (active): Phase IV to determine the efficacy of biennial screening with MRI in breast cancer Active: Phase II in diffuse pontine gliomas, malignant brain tumors, and stage IV renal cell carcinoma etc. Closed or completed: 35 clinical trials	[70]
	β-lapachone (ARQ 501)	ROS production	Completed: Phase II in pancreatic cancer (in combination with gemcitabine), metastatic leiomyosarcoma and metastatic squamous cell cancer of the head and neck; Phase I in combination with docetaxel in carcinoma	[71]
	STA-4783 (Elesclomol sodium)	ROS production	Active: Phase I in relapsed or refractory acute myeloid leukemia; Phase II in ovarian epithelial, fallopian tube, and primary peritoneal cancers Closed (temporarily): Phase I/II in metastatic prostate cancer (solid tumors)	[72,73]
	Arsenic trioxide (Trisenox)	ROS production, ANT ligand	Active: Phase IV in relapsed promyelocytic leukemia etc. (13 ongoing clinical trials) Closed or completed: 60 clinical trials	[74]
	DMAPT	ROS production	Discovery	[75]
	Parthenolide	ROS production	Discovery	[76]
	Bistetrahydrofuranic acetogenins	ROS production	Discovery	[77]
